# Incidence of Inadvertent Intraoperative Hypothermia and Its Risk Factors in Patients Undergoing General Anesthesia in Beijing: A Prospective Regional Survey

**DOI:** 10.1371/journal.pone.0136136

**Published:** 2015-09-11

**Authors:** Jie Yi, Ziyong Xiang, Xiaoming Deng, Ting Fan, Runqiao Fu, Wanming Geng, Ruihong Guo, Nong He, Chenghui Li, Lei Li, Min Li, Tianzuo Li, Ming Tian, Geng Wang, Lei Wang, Tianlong Wang, Anshi Wu, Di Wu, Xiaodong Xue, Mingjun Xu, Xiaoming Yang, Zhanmin Yang, Jianhu Yuan, Qiuhua Zhao, Guoqing Zhou, Mingzhang Zuo, Shuang Pan, Lujing Zhan, Min Yao, Yuguang Huang

**Affiliations:** 1 Peking Union Medical College Hospital, Beijing, China; 2 3M China R&D Center, Shanghai, China; 3 Plastic Surgery Hospital and Institute, CAMS, PUMC, Beijing, China; 4 Tsinghua University Yuquan Hospital, Beijing, China; 5 Beijing Chuiyangliu Hospital, Beijing, China; 6 Beijing Chest Hospital, Capital Medical University, Beijing, China; 7 Miyunxian Hospital, Beijing, China; 8 Peking University Shougang Hospital, Beijing, China; 9 China-Japan Friendship Hospital, Beijing, China; 10 China Meitan General Hospital, Beijing, China; 11 Peking University Third Hospital, Beijing, China; 12 Beijing Tongren Hospital Capital Medical University, Beijing, China; 13 Beijing Friendship Hospital, Capital Medical University, Beijing, China; 14 Beijing Jishuitan Hospital, Beijing, China; 15 Haidian Maternal & Child Health Hospital, Beijing, China; 16 Xuanwu Hospital Capital Medical University, Beijing, China; 17 Beijing Chao-Yang Hospital, Beijing, China; 18 Luhe Teaching Hospital of the Capital Medical University, Beijing, China; 19 Air Force General Hospital, PLA, Beijing, China; 20 Beijing Obstetrics and Gynecology Hospital, Capital Medical University, Beijing, China; 21 Central Hospital of China Aerospace Corporation, Beijing, China; 22 Beijing Rectum Hospital, Beijing, China; 23 Beijing Shi Jing Shan Hospital, Beijing, China; 24 Beijing Pinggu Hospital, Beijing, China; 25 Beijing Hospital of the Ministry of Health, Beijing, China; Massachusetts General Hospital, UNITED STATES

## Abstract

**Background/Objective:**

Inadvertent intraoperative hypothermia (core temperature <36^0^ C) is a recognized risk in surgery and has adverse consequences. However, no data about this complication in China are available. Our study aimed to determine the incidence of inadvertent intraoperative hypothermia and its associated risk factors in a sample of Chinese patients.

**Methods:**

We conducted a regional cross-sectional survey in Beijing from August through December, 2013. Eight hundred thirty patients who underwent various operations under general anesthesia were randomly selected from 24 hospitals through a multistage probability sampling. Multivariate logistic regression analyses were applied to explore the risk factors of developing hypothermia.

**Results:**

The overall incidence of intraoperative hypothermia was high, 39.9%. All patients were warmed passively with surgical sheets or cotton blankets, whereas only 10.7% of patients received active warming with space heaters or electric blankets. Pre-warmed intravenous fluid were administered to 16.9% of patients, and 34.6% of patients had irrigation of wounds with pre-warmed fluid. Active warming (OR = 0.46, 95% CI 0.26–0.81), overweight or obesity (OR = 0.39, 95% CI 0.28–0.56), high baseline core temperature before anesthesia (OR = 0.08, 95% CI 0.04–0.13), and high ambient temperature (OR = 0.89, 95% CI 0.79–0.98) were significant protective factors for hypothermia. In contrast, major-plus operations (OR = 2.00, 95% CI 1.32–3.04), duration of anesthesia (1–2 h) (OR = 3.23, 95% CI 2.19–4.78) and >2 h (OR = 3.44, 95% CI 1.90–6.22,), and intravenous un-warmed fluid (OR = 2.45, 95% CI 1.45–4.12) significantly increased the risk of hypothermia.

**Conclusions:**

The incidence of inadvertent intraoperative hypothermia in Beijing is high, and the rate of active warming of patients during operation is low. Concern for the development of intraoperative hypothermia should be especially high in patients undergoing major operations, requiring long periods of anesthesia, and receiving un-warmed intravenous fluids.

## Introduction

Intraoperative hypothermia, defined as core temperature <36°C during operation, is a common problem among surgical patients [1,2]. An incidence of the condition of 4% to 72% [3–5], and up to 90% in some studies [6,7], has been reported. Many professional societies, such as the Association of periOperative Registered Nurses (AORN), www.aron.org, and the National Institute for Health and Care Excellence (NICE), www.nice.nhs.uk [8], have made recommendations for preventing hypothermia and improving its management during the perioperative period. Intraoperative hypothermia has been associated with numerous complications, including risk of cardiovascular diseases [9,10], perioperative hemorrhage [11,12], disturbed drug metabolism [13], and postoperative infection [14,15] Many factors may contribute to an increased risk of hypothermia: impairment of thermoregulation by general anesthesia, low temperature in the operating room, and the use of un-warmed fluids for intravenous infusion or wound irrigation.

Although many studies of inadvertent intraoperative hypothermia have been reported from western countries, similar studies have not been conducted in China, the largest patient population in the world. The use of patient warming during operation and the risk factors of inadvertent intraoperative hypothermia in China have not been examined. Since inadvertent intraoperative hypothermia is a modifiable condition, understanding these factors may help prevent the hypothermia and its associated complications. Therefore, we have conducted this cross-sectional multi-center survey in Beijing in order to ascertain the incidence of inadvertent intraoperative hypothermia and its risk factors in a Chinese population.

## Patients and Methods

### Study sample

This is a cross sectional, multi-center study, which started in August, 2013 and ended in December, 2013. As an observational study, this study protocol was fully approved first by the Ethics Committee and Institutional Review Board (IRB) by Peking Union Medical College Hospital (PUMCH), the leading site of the entire study. Then the protocol was accepted by all other participating sites, which were Plastic Surgery Hospital and Institute; Tsinghua University Yuquan Hospital; Beijing Chuiyangliu Hospital; Beijing Chest Hospital; Miyunxian Hospital; Peking University Shougang Hospital; China-Japan Friendship Hospital; China Meitan General Hospital; Peking University Third Hospital; Beijing Tongren Hospital; Beijing Friendship Hospital; Beijing Jishuitan Hospital; Haidian Maternal and Child Health Hospital; Beijing Xuanwu Hospital; Beijing Chao-Yang Hospital; Luhe Teaching Hospital; Air Force General Hospital PLA; Beijing Obstetrics and Gynecology Hospital; Central Hospital of China Aerospace Corporation; Beijing Rectum Hospital; Beijing Pinggu Hospital; and Beijing Hospital of the Ministry of Health. This study had been also registered at www.clinicaltrial.gov *(National Clinical Trial (NCT) number*: *NCT01913041*). All participants were required to sign informed consent forms before being enrolled in the study.

The study included subjects from 24 hospitals in Beijing who underwent elective operations with an estimated duration of general anesthesia of more than 30 minutes. Participants were excluded if they had: 1) high central fever caused by cerebrovascular disease, cerebral trauma, cerebral operations, epilepsy, or acute hydrocephalus; 2) thermoregulation abnormalities (e.g., malignant hyperthermia, neuroleptic malignant syndrome); 3) infectious fever with core temperature one week before operation higher than 38.5°C; or 4) history of hypothyroidism or hyperthyroidism. Subjects also were excluded if they were younger than 18 years of age (because of parents’ concerns for the risk of tympanic membrane perforation during temperature monitoring).

### Multistage random sampling

To derive the sampling size needed to detect a statistically significant difference, the sample size was estimated by N = PQ/ (d/t)^2^, where P was denoted as the incidence of hypothermia; Q = 1-P; d was an acceptable error (usually 10% of P); t was a statistic for the significance test. The incidence of hypothermia P was set at 0.45. Because of the lack of data in China, the incidence of intraoperative hypothermia was estimated as 46%, based on previous studies [16]. Thus, 489 study subjects were required in this study. Considering cluster sampling and the balance over various hospitals [17], an additional 50% was added to the study population; thus the planned total enrollment was 800 subjects.

In the first stage, investigators obtained a full list of 70 hospitals from City of Beijing Health Administration Bureau. These hospitals were either academic teaching hospitals or local community hospitals. Cosmetic and dental clinics were excluded. Using a simple randomization, 24 hospitals were selected from 70 hospitals. In the second stage, at each participating hospital site, investigator identified all eligible patients from a list of scheduled surgeries (issued by OR daily) one day prior to the surgery day. This name list of eligible patients was then submitted to statistician for randomization. Our statistician randomly selected two patients through a computer program for a site investigator every day. Site investigator then contacted selected patients for their consent. Patients were enrolled after they agreed and signed informed consent forms. Thus, 830 subjects were included in the analyses.

### Intraoperative hypothermia and core temperature

The primary outcome was inadvertent intraoperative hypothermia, defined as core temperature <36°C at any time during the perioperative period. The core temperature was assumed to be that of tympanic membrane temperature, since tympanic membrane temperature is easily obtained and has been validated to reflect core temperature [15]. We applied a tympanic membrane thermometer (ThermoScan PRO-4000, Braun GmbH, Kronberg, Germany) to monitor temperature every 15 minutes before, during, and after operation. To reduce bias, the thermometer was calibrated and validated according to the manufacturer’s manual before use.

### Anesthesia and other risk factors

All patients received either general anesthesia only or general anesthesia combined with regional anesthesia, depending on the preference of each hospital. The regimens of general anesthesia used were mostly propofol (2–2.5 mg/kg), fentanyl (2–4 μg/kg), and rocuronium (0.8–1 mg/kg) as induction, and sevoflurane (1.5–2 vol %) mixed with O_2_/N_2_O (50%/50%) for maintenance. Ropivacaine or lidocaine was used for regional anesthesia.

Patients’ demographic data were collected: age, gender, body mass index (BMI), medical history, and American Society of Anesthesiologists (ASA) physical status. BMI was calculated as the weight in kilograms divided by the squared height in meters; ≥25 km/m^2^ was considered overweight or obese. The following data, considered potential risk factors, also were collected: baseline core temperature (prior to anesthesia), ambient temperature of the operating room, types of patient warming, amount of intravenous fluid replacement, duration of anesthesia, and magnitude of the operation. Kinds of patient warming were categorized as passive (comforter, blanket, etc.) or active (electric heated blanket, space heater, etc.). The magnitude of operations was classified according the classification used in a previous study [17]: a) minor surgery (e.g., excision of cutaneous lesion, drainage of breast abscess); b) intermediate surgery (e.g., primary repair of inguinal hernia, excision of varicose vein(s) of the leg, tonsillectomy/adenotonsillectomy, or knee arthroscopy; c) major surgery (e.g., total abdominal hysterectomy, endoscopic resection of the prostate, lumbar discectomy, or thyroidectomy); and d) major surgery-plus (e.g., total joint replacement, lung operations, colonic resection, radical neck dissection, neurosurgery, or cardiac surgery).

### Statistical analysis

Descriptive analysis, including mean, standard deviation, frequencies and percentages were presented, respectively. Multivariate logistic regression was applied to evaluate the potential risk factors for inadvertent intraoperative hypothermia. The results were presented as odds ratios together with a 95% confidence interval (95% CI). All analyses were performed with SAS 9.0 (SAS Institute, Cary, NC).

## Results

### Demographics of the study population

Eight hundred thirty subjects from 24 hospitals in Beijing were enrolled (Table 1). The mean age of the study population was 50.1 ± 16.9 years, and two-thirds of the subjects were male. BMI was 24.7±7.2 kg/m^2^. The majority of subjects was either ASA I (33.8%) or ASA II (56.1%) class. General and gynecologic operations were the two most frequent operations: 28.2% and 27.4%, respectively. Forty-seven (5.7%) subjects received general anesthesia combined with regional blockage. Major surgery constituted 59.7% of the operations, and major-plus surgery 23.8%. Operation time was 2.1±1.4 h and mean anesthesia time 2.5±1.5 h.

**Table 1:** Patient Demographics and Anesthesia/Surgery Data, Beijing, China (N = 830).

Variables	Value
**Age, n, mean± std (yr)**	830, 50.1±16.9
**Age>65, n (%)**	107 (12.9)
**Gender, n (%)**	830 (100.0)
Male	500 (60.2)
Female	330 (39.8)
**BMI, n, mean± std**	830, 24.7±7.2
**History, n (%)**	830 (100.0)
Smoking	152 (18.3)
Alcohol	109 (13.1)
Hypertension	225 (27.1)
Cerebral Vascular disease	42 (5.1)
Cardiovascular disease	272 (32.8)
Diabetes	80 (10.0)
Chronic liver disease	35 (4.2)
**ASA, n (%)**	830 (100.0)
1	282 (33.8)
2	468 (56.1)
3	78 (9.4)
4	4 (0.5)
**Type of Surgery, n (%)**	830 (100.0)
General Surgery	234 (28.2)
OB/GYN	227 (27.4)
Perpherial Vasular Surgery	12 (1.5)
Cardiovascular Surgery	8 (0.9)
Thoracic Surgery	70 (8.4)
Orthopaedics Surgery	126 (15.2)
Neurosurgery	22 (2.7)
Uroloy	72 (8.7)
Plastic Surgery	27 (3.3)
Others	32 (3.9)
**Magnitude of Surgery** ^**1**^ **, n (%)**	830 (100.0)
minor	5 (0.6)
intermediate	169 (20.4)
major	421 (50.7)
major plus	235 (23.8)
**Endoscopic Surgery** ^**2**^ **, n (%)**	427 (51.5%)
**Mode of Anesthesia, n (%)**	830 (100.0)
General	783 (94.3)
Combined general	47 (5.7)
**Total Surgery Time** ^**3**^ **, n, mean± std (h)**	830, 2.1±1.4
**Total Anesthesia Time** ^**4**^ **, n, mean± std (h)**	830, 2.5±1.5

^1^ Magnitude of surgery as below: minor surgery: excision of lesion of skin; drainage of breast abscess, etc. Intermediate surgery: primary repair of inguinal hernia; excision of varicose vein(s) of leg; tonsillectomy/adenotonsillectomy; knee arthroscopy, etc. major surgery: total abdominal hysterectomy; endoscopic resection of prostate; lumbar discectomy; thyroidectomy, etc. major surgery plus: total joint replacement; lung operations; colonic resection; radical neck dissection; neurosurgery; cardiac surgery)

^2^ Arthroscopic, laparoscopic, etc

^3^ Time from incision to closure

^4^ Time from induction to discontinuation of anesthetic agents

### Incidence of hypothermia and core temperature

**Fig 1:**
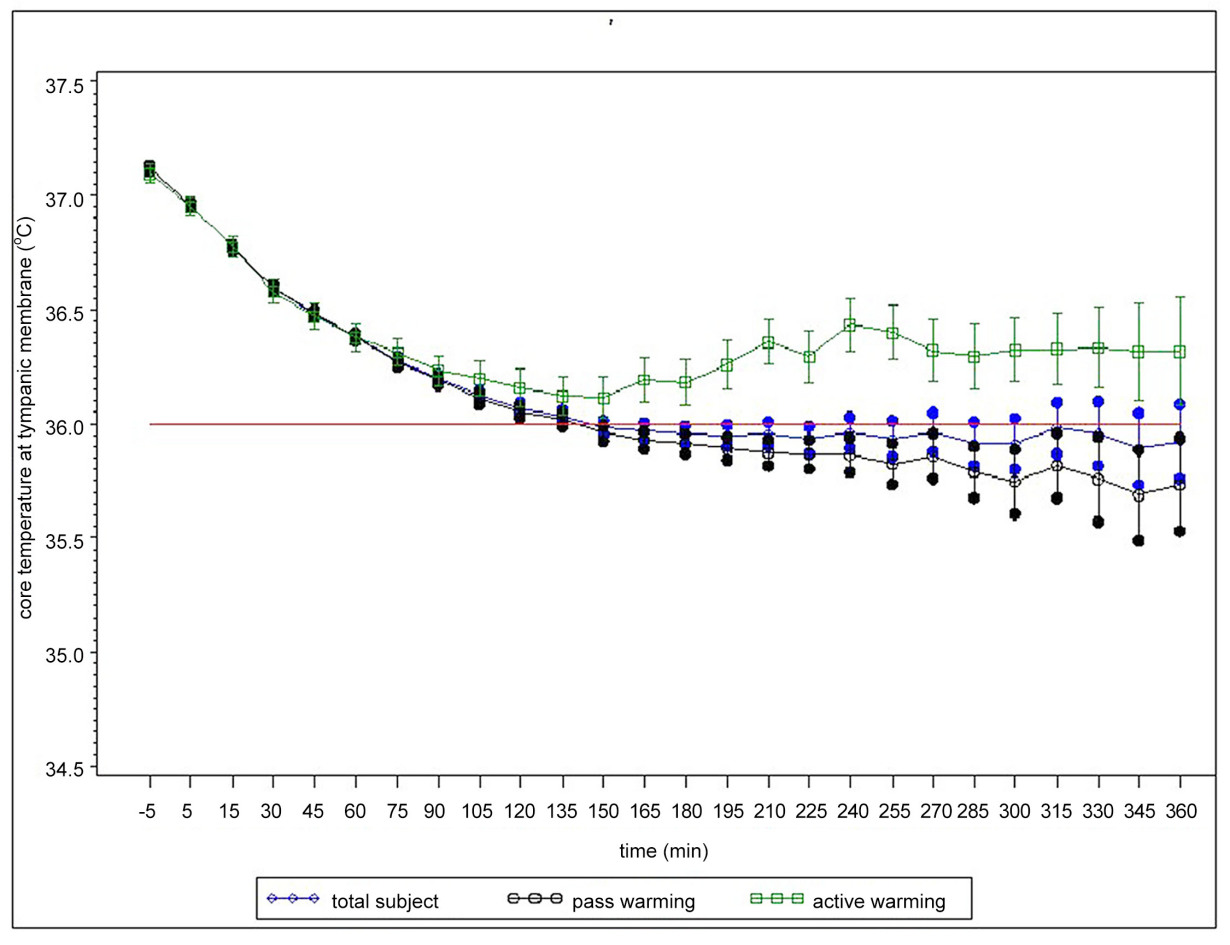
Change of intraoperative core temperature during operations. Patients’ core temperature was measured at the tympanic membrane beginning every 15 minutes after the induction of anesthesia and until the end of the operation. A total of 830 subjects were enrolled in the study; 89 (10.7%) received active warming, and 741(89.3%) received no warming.

The overall incidence of inadvertent intraoperative hypothermia was 39.9%. The incidence was 17.1% in operations of less than 2 hours, and 44.8% in operations of more than two hours. All patients received passive warming, and 10.7% received active warming (Table 2). The core temperature of patients with active warming remained above 36°C throughout the operation, whereas the core temperature of patients with passive warming fell below 36°C after an average of 135 min operation time. The mean difference of core temperature between the two groups began to increase one hour after induction of anesthesia (Fig 1).

**Table 2:** Incidence of intraoperative hypothermia, patient warming and clinical outcome (N = 830).

Variables	Value
**Overall Incidence of Hypothermia**	
T< 36.0°C, % (95%CI)	39.9% (36.6–43.2%)
T<35.5°C, % (95%CI)	16.9% (14.2–19.5%)
T<35.0°C, % (95%CI)	6.1% (4.5–7.8%)
**Incidence of hypothermia within 2 hours**	17.1%
**Incidence of hypothermia beyond 2 hours**	44.8%
**Baseline core temperature prior to anesthesia (°C), mean±std**	37.1±0.4
Baseline core temperature = 36.0°C, n (%)	3 (0.4)
36.0°C <Baseline core temperature ≤36.5°C, n (%)	50 (6.0)
36.5°C <Baseline core temperature ≤37.0°C, n (%)	288 (34.7)
Baseline core temperature >37.0°C, n (%)	489 (58.9)
**OR ambient temperature (°C), mean±std**	23.5±1.7
**Patient receiving intraoperative passive warming** ^**1**^ **, n (%)**	830 (100.0)
**Patient receiving intraoperative active warming** ^**2**^ **, n (%)**	89 (10.7)
space heater	18 (20.2)
space heater blanket	10 (11.2)
electric blanket	31 (34.8)
water heated blanket	30 (33.7)
**Blood transfusion**	
autologous	
n (%)	52 (6.3)
mean±std (ml)	429.9±259.3
median (ml)	400
min-max (ml)	100–1250
pre-warmed, n (%)	5 (9.62)
allogeneic	
n (%)	52 (6.3)
mean±std (ml)	791.4±777.2
median (ml)	800
min-max (ml)	150–5800
pre-warmed, n (%)	52 (100)
**Perioperative IV fluid**	
mean±std (ml)	1702.5±914.9
median (ml)	1500
min-max (ml)	200–7300
**Total patients receiving IV fluid replacement**	
patients receiving prewarmed IV fluid, n (%)	141 (16.9)
patients receiving unwarmed IV fluid, n (%)	689 (83.0)
**Intraoperative irrigation fluid**	
mean±std (ml)	650.4±975.4
median (ml)	300
min-max (ml)	0–7000
**Total patients receiving intraopertive irrigation fluid, n (%)**	480 (100.00)
patients receiving prewarmed irrigation fluid, n (%)	166 (34.6)
patients receiving unwarmed irrigation fluid, n (%)	314 (65.4)
**Level of shiver, n (%)**	
No	707 (85.2)
Yes	123 (14.8)

^1^ passive warming includes comforter, blanket etc

^2^ active warming includes electric heated blanket, space heater etc

The amount of intraoperative fluid replaced was 200–7300 ml (median 1500 ml), including crystal and colloid fluids. Eighty-three per cent of patients received infusion of un-warmed fluids. About 9.6% of patients were transfused with pre-warmed autologous blood, and all patients who were transfused with allogeneic blood received pre-warmed blood. For intraoperative irrigation of wounds, 34.6% of patients received pre-warmed fluids. After being transferred to the post-anesthesia care unit, 14.8% of patients experienced shivering (Table 2).

### Risk factors of hypothermia

Patients who received active warming were 0.46 times as likely to develop hypothermia during the operation as patients who received passive warming (OR = 0.46, 95% CI 0.26–0.81). Overweight (BMI ≥25) (OR = 0.39, 95%CI 0.28–0.56), elevation of baseline core temperature prior to anesthesia (OR = 0.08, 95%CI 0.04–0.13), and high ambient temperature (OR = 0.89, 95%CI 0.79–0.98) also were protective factors of hypothermia. In contrast, major-plus surgery (OR = 2.00, 95%CI 1.32–3.04); duration of anesthesia 1–2 h (OR = 3.23, 95% CI 2.19–4.78) and >2 h (OR = 3.44, 95%CI 1.90–6.22); and infusion of intravenous un-warmed fluid (>1000 ml) (OR = 2.45, 95%CI 1.45–4.12) significantly increased the incidence of intraoperative hypothermia (Table 3).

**Table 3:** Risk factors associated with intraoperative hypothermia, Beijing, China (N = 830).

	Adjusted OR^1^ (95%CI)	P value
**Age**		
< = 65	reference	
>65	0.72 (0.42–1.23)	0.2243
**Gender**		
Male	reference	
Female	0.76 (0.53–1.09)	0.1387
**ASA**		
<3	reference	
≥3	1.01 (0.55–1.87)	0.9568
**Magnitude of Surgery**		
Minor/Intermediate/Major Surgery	reference	
Major Plus Surgery	1.93 (1.27–2.95)	0.0021
**Anesthesia**		
General Anesthesia alone	reference	
Anesthesia (combined)	1.84 (0.85–3.99)	0.1204
**IV fluid replacement** ^**2**^ **(>1000ml)**		
≤1000ml	reference	
>1000ml	2.78 (1.74–4.46)	<0.0001
**Intraoperative irrigation**		
≤1000ml	reference	
>1000ml	0.73 (0.47–1.15)	0.1806
**Duration of anesthesia**		
≤1h	reference	
1 to 2 h	3.08 (2.07–4.57)	0.0033
>2 h	3.284 (1.81–5.95)	0.0195
**Endoscopic Surgery**		
no	reference	
yes	0.76 (0.53–1.09)	0.1422
**Warming**		
passive warming	reference	
active warming	0.44 (0.25–0.78)	0.0049
**Baseline core temperature before anesthesia (C°)**	0.075 (0.04–0.13)	<0.0001
**BMI**		
≤25	reference	
>25	0.39 (0.28–0.56)	<0.0001
**Ambient temperature (C°)**	0.88 (0.79–0.98)	0.0244

OR, Odds Ratio, Significant level indicates as P<0.05.

^1^ adjusted OR were presented after adjusting all the variables in above table.

^2^ patients receiving unwarmed IV fluid only

## Discussion

In this study, the incidence of inadvertent intraoperative hypothermia of Chinese surgical patients in Beijing who received general anesthesia was high, about 40%. Major surgery, longer duration of anesthesia, and infusion of larger amounts (more than 1000 ml) of un-warmed intravenous fluids were risk factors for hypothermia.

Data on intraoperative hypothermia in China has been needed. However, to conduct a nationwide, large-scale, epidemiologic study without preliminary investigation would be difficult, given China’s geographic, cultural, and socioeconomic diversity. Thus, we conducted this initial, limited survey in Beijing. The study is one of several initiatives in a national campaign to improve perioperative patient safety and practice quality in China. We selected Beijing for this study because the city has some of the best health-care providers and resources in China. Also, clinical studies from Beijing usually have strong protocol compliance, study implementation, and data quality control.

The reported incidence of hypothermia varies widely, probably in part because of differences in definitions and times of recording hypothermia, which has varied from postoperative admission to the post-anesthesia care unit or intensive care unit [14,17–19], and to time of closure [5]. In order to avoid underestimation of the incidence of hypothermia, we recorded hypothermia at any point in the perioperative period [1,14]. Nevertheless, the incidence in our patients was lower than that reported by some [20,21], who found hypothermia in 65%-74% of patients undergoing major abdominal operations. This difference might be due to differences in the complexity of operations and duration of anesthesia. We did not include cardiovascular surgery patients in this study because some such patients receive therapeutic hypothermia. We also excluded pediatric patients because of their inadequate thermoregulatory response to hypothermia [22].

This study, a snapshot of current practices of perioperative core temperature management in Beijing hospitals, reveals some practice shortcomings. Only about 10% of the patients received active warming, with techniques such as forced-air warming systems. Most patients were passively warmed with cotton blankets, sheets, or surgical draping, but these measures have been reported ineffective in maintaining core temperature [23]. Although use of warmed fluids has been regarded beneficial in maintaining perioperative normothermia [24,25], un-warmed intravenous fluids were used in 83% of our patients and un-warmed irrigation fluids in 65%, which may have contributed to the frequency of hypothermia. Subjects who received more than 1 liter of un-warmed fluid had nearly a three-fold risk of hypothermia (OR = 2.67, 95%CI: 1.67–4.26) compared with subjects who received less than 1 liter. Barthel, et al [26] showed that infusion of 2 L of room-temperature crystalloid in the average normothermic adult resulted in a decrease in body temperature of about one-third degree Celsius. The prevailing evidence indicates that the use of un-warmed fluids should be minimized, whereas pre-warmed or warmed fluids can help prevent hypothermia [27,28].

Clinical variables associated with perioperative hypothermia have differed among studies [29–31]. In this study, major-plus surgery and duration of anesthesia, either 1–2 hours or longer than 2 hours, were significant risk factors in predicting hypothermia, a finding consistent with those of a previous study [32] but different from others’ findings [33,34]. In this study, multivariate analysis suggested that the combination of epidural or regional anesthesia with general anesthesia increased the risk of hypothermia, but the results did not reach statistical significance, perhaps because of the small sample size of combined anesthesia (47 patients). The results of earlier studies [21,35,36] indicated that older patients may be predisposed to hypothermia, but results of our study differed, perhaps because the cutoff age was 65 years and the number of subjects older than that was too small (107 cases, 13.0%).

In this study, the protective factors of hypothermia were BMI >25, relatively high ambient temperature, and patients’ core temperature before anesthesia, and active warming. Although only a small proportion of our patients (10.7%) received active warming, the warming was a strong, statistically significant risk factor against hypothermia (P = 0.0072). This result in a Chinese population is in agreement with the results in other populations [6].

We believe this is the first study of inadvertent perioperative hypothermia rates in China. The survey evaluates the incidence and clinical risk factors in a prospective manner. However, there are several limitations to this study. First, although the study population well represents the Beijing region, it may not represent other cities or areas in China, given the country’s remarkable regional differences. A nation-wide epidemiologic survey is currently in progress based on the results of this pilot study. Second, the frequency of measuring patient’s core temperature in ordinary operating-room practice may differ from the frequency in this study; thus it is possible that intraoperative hypothermia is more common in usual practice than we recorded. Finally, the postoperative outcome of intraoperative hypothermia, such as incidence of wound infection and cardiovascular events, is unknown.

## Conclusion

This study revealed a high incidence of inadvertent hypothermia in a sample of Chinese surgical patients (Beijing) and identified risk factors associated with the hypothermia. A relatively high incidence of hypothermia was found in patients undergoing major-plus surgery, receiving more than 1L of un-warmed intravenous fluid, and undergoing lengthy procedures. The rate of active warming of patients during operation was low. Thus, the study reveals risk factors that should be heeded and measures to be taken in order to prevent intraoperative hypothermia. Finally, study of intraoperative hypothermia in China should be extended to a larger spectrum of the population.

## Supporting Information

S1 STROBE Checklist STROBE Checklist. (DOCX)

S1 Protocol Study Protocol (in Chinese). (DOCX)

S2 Protocol Study Protocol (English translation). (DOCX)
