# Supplementary material for: Incidence of Inadvertent Intraoperative Hypothermia and Its Risk Factors in Patients Undergoing General Anesthesia in Beijing: A Prospective Regional Survey
Source: PLoS One. 2015 Sep 11;10(9):e0136136. doi: 10.1371/journal.pone.0136136 (PMC4567074; doi:10.1371/journal.pone.0136136)
Supplement: S1 Protocol — (DOCX) [file pone.0136136.s002.docx]

方案编号：MDI0077

**北京地区择期全麻患者围术期低体温发生率**

**流行病学调查**

| **组长单位：** | **中国医学科学院北京协和医院麻醉科** |
| --- | --- |
| **主要研究者:** | **黄宇光** |
| **主要协调研究者** | **易杰** |
| **方案版本日期:** | **2013年6月30日** |

目 录

[研究摘要 3](#_Toc363059796)

[1. 研究背景 6](#_Toc363059797)

[2. 研究目的 6](#_Toc363059798)

[3. 研究设计及研究期限 6](#_Toc363059799)

[4. 研究的暴露因素 6](#_Toc363059800)

[4.1. 病人危险因素 6](#_Toc363059801)

[4.2. 麻醉危险因素 6](#_Toc363059802)

[4.3. 手术及其它危险因素 7](#_Toc363059803)

[4.4. 其它危险因素 7](#_Toc363059804)

[5. 研究的主要临床结局 7](#_Toc363059805)

[6. 研究研究方法及技术路线 7](#_Toc363059806)

[6.1. 选择符合研究要求的研究现场及研究对象 7](#_Toc363059807)

[6.1.1. 研究现场：本研究拟选取北京市2级甲等以上医院 7](#_Toc363059808)

[6.1.2. 研究对象：调查期间接受择期全身麻醉手术的患者 7](#_Toc363059809)

[6.2. 基线及术前准备期 8](#_Toc363059810)

[6.3. 术中和术后 8](#_Toc363059811)

[7. 研究统计学考虑及统计分析计划 9](#_Toc363059812)

[7.1. 样本量计算和抽样方法 9](#_Toc363059813)

[7.1.1. 样本量计算 9](#_Toc363059814)

[7.1.2. 抽样方法：采用多级抽样方法 9](#_Toc363059815)

[7.1.3. 总体患者基线数据收集： 10](#_Toc363059816)

[7.2. 数据管理和统计分析 12](#_Toc363059817)

[7.2.1. 数据管理 12](#_Toc363059818)

[7.2.2. 统计分析 12](#_Toc363059819)

[8. 受访者完成及退出研究 13](#_Toc363059820)

[8.1. 受访者完成研究 13](#_Toc363059821)

[8.2. 受访者退出研究 13](#_Toc363059822)

[8.3. 筛选和基线评估失败 13](#_Toc363059823)

[9. 质量控制与保证 13](#_Toc363059824)

[9.1. 方案偏离 13](#_Toc363059825)

[9.2. 培训 14](#_Toc363059826)

[9.3. 法规依从性 14](#_Toc363059827)

[9.4. 研究监查 14](#_Toc363059828)

[9.4.1. 研究中心的监查 14](#_Toc363059829)

[9.4.2. 依从性评估 14](#_Toc363059830)

[10. 数据 14](#_Toc363059831)

[10.1. 源文件 14](#_Toc363059832)

[11. 病例报告表的完成 15](#_Toc363059833)

[12. 伦理学考虑 15](#_Toc363059834)

[12.1. 伦理委员会审查 15](#_Toc363059835)

[12.2. 知情同意 15](#_Toc363059836)

[12.3. 保密性 15](#_Toc363059837)

[13. 技术路线图 16](#_Toc363059838)

[14. 计划进度 16](#_Toc363059839)

[14.1. 研究持续时间 16](#_Toc363059840)

[14.2. 研究进度 16](#_Toc363059841)

[附录A 17](#_Toc363059842)

[附录B 18](#_Toc363059843)

[附录C 19](#_Toc363059844)

[附录D 20](#_Toc363059845)

[附录E 21](#_Toc363059846)

研究摘要

| 研究题目： | 北京地区择期全麻患者围术期低体温发生率流行病学调查 |
| --- | --- |
| 研究目的： | - 了解和掌握目前北京地区择期全麻手术围术期低体温发生率 - 对调查资料进行亚组分析，探索低体温发生的高危因素 |
| 研究现场： | 北京二级以上医院(拟随即抽取8家二级医院和16家三级医院) |
| 目标人群： | 接受全麻的择期手术患者 |
| 入选标准 | - 男性或女性，年龄不限 - 接受全身麻醉的择期手术患者 - 预计手术时间持续超过40分钟 - 获取知情同意 |
| 排除标准 | - 中枢性高热患者，包括脑血管病，脑外伤和脑手术，癫痫，急性脑积水引起的中枢性高热，明确证据确诊的甲减和甲亢病人 - 体温调节异常包括恶性高热（MHS）和神经安定剂恶性综合征 - 感染性发热 - 术前1周内体温高于38.5摄氏度 - 耳道感染等影响耳鼓膜温度准确测定的疾病和手术 - 术中采取主动降温措施的手术 |
| 样本总量： | 基于文献报道的低体温发生率约为45 %，根据现象调查的样本量估计公式N=PQ/(d/t)2，P为低体温发生率，取值0.45，Q=1-P=0.55，d为可容许误差，一般取P值的10%左右，t为显著性检验的统计量，估计所需患者489例。考虑到是整群抽样及各个医院之间的均衡，在估计样本的基础上，再增加50%，拟入组800名受访者。 |
| 抽样方法： | 多级抽样方法(分层+整群+随机抽样) |
| 主要观察指标： | 低体温发生率 |
| 统计分析计划 | 1. 低体温发生率：发生低体温的患者数/被调查的总数×100%。2. 多因素分析：采用Logistic多元回归分析，对单因素的高危因素进行多元回归分析，确定各高危因素的影响程度。3. 术后体温变化的曲线拟合的统计学建模。 |

**全部参与单位**

| **中心编号** | **单位名称** |
| --- | --- |
| **01** | **北京协和医院** |
| **02** | **空军总医院** |
| **03** | **首都医科大学附属北京友谊医院** |
| **04** | **航天中心医院(原721医院)** |
| **05** | **北京市密云县医院** |
| **06** | **北京市垂杨柳医院** |
| **07** | **卫生部北京医院** |
| **08** | **清华大学玉泉医院** |
| **09** | **北京大学首钢医院** |
| **10** | **北京石景山医院** |
| **11** | **首都医科大学附属北京妇产医院** |
| **12** | **卫生部中日友好医院** |
| **13** | **北京积水潭医院** |
| **14** | **煤炭总医院** |
| **15** | **北京大学第三医院** |
| **16** | **北京市通州区潞河医院** |
| **17** | **首都医科大学宣武医院** |
| **18** | **首都医科大学附属北京同仁医院** |
| **19** | **北京市平谷区医院** |
| **20** | **中国医学科学院肿瘤医院** |
| **21** | **海淀区妇幼保健医院** |
| **22** | **北京胸科医院** |
| **23** | **北京肛肠医院(二龙路医院)** |
| **24** | **北京朝阳医院** |
| **25** | **整形外科医院** |

**研究者方案签字页**

我同意：

- 承担在本中心正确执行本临床研究的责任。
- 按照北京协和医院提供的方案及任何未来的修改案及实施程序来进行本研究。
- 未经申办者的同意及机构审查委员会或独立伦理委员会的审查和书面批准，不对方案执行任何修改，除非有必要避免对受访者的直接伤害或因为研究管理方面的要求（为所有适用的法律法规要求所允许的要求）。
- 我完全熟悉方案中和其他申办者提供的资料中所描述的研究产品的正确使用方法,其他资料包括但不局限于：最新的使用说明书或其等效文件。
- 我了解并将遵守“临床研究管理规范”（GCP）和所有适用法律法规的要求。
- 确保所有协助我参与本研究的人员充分了解本方案中描述的研究相关人员的职责。

| 研究者姓名（正楷）： |  |
| --- | --- |
| 研究者签字： |  |
| 签字日期： |  |

# 研究背景

轻度围术期低体温是很常见的现象，其发生比例约占所有手术病人的一半^[1]^，这与以下三个主要的不良事件直接相关并使风险显著增加:1）心脏并发症^[2, 3]^，2）出血^[4]^，3）感染并发症^[5, 6]^，尤其是在高风险患者（ASA III、IV级）^[7]^。此外，体温降低也可能导致以下两种情况：1）麻醉后恢复室（PACU）内滞留时间延长^[8]^，2）患者热舒适度下降^[9]^，从而影响了患者的满意度且增加了费用^[10]^。

从病人安全性角度看，体表温度不仅仅是一个生命体征^[11]^。因此，为预防围术期低体温，必须从手术最开始时就对核心温度进行监测。其次，术中热量损失应被最小化。再次，应在手术全程对高危患者给予主动升温措施^[12]^。

尽管国外文献报道围术期低体温发生率高，但国内尚无详细资料报道。目前国内已有体温保护的个案报道，但系统性体温保护策略的相关研究较少，亟待补充和完善有关的临床证据。全国性的和地区性的低体温发生率流行病学调查有待进一步加强，以完善相关统计学数据。

为了获得更多关于中国体温监测和病人保暖实践的信息，我们将对一个北京地区麻醉科的样本进行此次前瞻性调查。此研究将调查该地区围术期低体温发生率，探索则其全麻围术期低体温发生的原因，以及在此基础上，对围术期低体温的发生率开展预测，为采取术中保暖措施提供依据。

# 研究目的

1. 了解和掌握目前北京地区围术期低体温发生率。
2. 对调查资料进行亚组分析，探索低体温发生的高危因素。
3. 对受访者麻醉后的体温变化趋势进行曲线拟合。

# 研究设计及研究期限

此研究为北京地区择期全麻手术患者围术期低体温发生率的横断面研究。研究观察期为预先选定的两个月，在该期限内随机抽取需要观察的病例并收集相应的资料。

# 研究的暴露因素

## 病人危险因素

年龄（<14岁和>60岁），ASA分级>II级，BMI，体表面积，糖尿病，病人术前基础核心体温

## 麻醉危险因素

全麻，全麻复合其他麻醉，麻醉剂的种类，麻醉持续时间，麻醉平面

## 手术及其它危险因素

手术分级，手术持续时间（2小时），手术类型（内窥镜手术，非内窥镜手术），手术室温度

## 其它危险因素

术中输液量以及是否加温后使用，术中冲洗液量/输血量以及是否加温后使用，是否采取保温措施及何种保暖措施

# 研究的主要临床结局

1. 全部调查患者及亚组的术后低体温发生率。
2. 全部调查患者及亚组的术前，术中（麻醉开始后每隔15分钟记录一次），以及术后（进ICU/病房前）低体温发生率。

低体温定义为核心体温温度在36°C以下：

- 轻度： 核心体温： 35- 35.9°C
- 中度： 核心体温： 34- 34.9°C
- 重度： 核心体温： ≤ 33.9°C

*低体温发生率：*发生低体温的患者数/被调查的总数×100%

1. 术后睁眼时间
2. 术后PACU滞留时间及内寒颤发生率
3. ICU住院时间和总住院时间
4. 术后30天死亡率
5. 术后30天手术切口感染率

# 研究研究方法及技术路线

## 选择符合研究要求的研究现场及研究对象

### 研究现场：本研究拟选取北京市2级甲等以上医院

### 研究对象：调查期间接受择期全身麻醉手术的患者

1. 入选标准

- 男性或女性，年龄不限
- 接受全身麻醉的择期手术患者
- 预计手术时间持续超过40分钟
- 获取知情同意

1. 排除标准

- 中枢性高热患者，包括脑血管病，脑外伤和脑手术，癫痫，急性脑积水引起的中枢性高热，明确证据确诊的甲减和甲亢病人
- 体温调节异常包括恶性高热（MHS）和神经安定剂恶性综合征
- 感染性发热
- 术前1周内患者核心体温超过38.5°C
- 耳道感染等影响耳鼓膜温度准确测定的疾病和手术
- 术中采取主动降温措施的手术

## 基线及术前准备期

在进行该研究之前，须按照GCP的规定，对符合入排标准的受访者进行知情同意过程，并获得该患者知情同意签字。

基线时应收集以下数据：

- 人口统计学数据，包括：年龄、性别和种族
- 病史回顾，体格检查，包括身高和体重，ASA分级
- 进入手术等侯区时的核心体温（若无手术等候区域则采集患者进入手术室前30分钟内核心体温）

进入手术室后的准备期：

- 手术室温度
- 术前是否采取预保暖措施，所采取保暖措施的类型及持续时间

## 术中和术后

患者应依据中国医疗实践标准接受适当的治疗。

需收集以下数据：

- 麻醉的类型，麻醉、镇痛和镇静剂的剂量，麻醉开始，结束时间
- 手术类型，手术名称，手术分级，手术持续的时间，体腔暴露时间，体腔冲洗液的使用量及是否加热，静脉输液量及是否加热，输血量及是否加热，术中总出量及总入量
- 手术室温度，术中采取的保暖措施及持续时间
- 每15分钟测量和记录耳鼓膜温度1次（其中包括麻醉诱导前5分钟和麻醉诱导后5分钟，视窗时间3分钟）
- 每5分钟测量和记录鼻咽部温度1次（视窗时间为1分钟）

**麻醉后护理室（PACU）**

手术结束后，患者被转移到恢复区，此期间研究人员记录麻醉后恢复时间、寒战、恶心和呕吐。患者可转出恢复室的参考标准如下：

- 患者有意识和反应，疼痛和恶心症状已得到控制
- 生命体征平稳（收缩压在基线水平的20%以内，心率范围在50到100次/分）
- 在未输血的情况下血红蛋白浓度稳定
- 自主呼吸时外周血氧饱和度超过95%
- 中心温度≥36°C

*记录达到以上转出恢复室标准的时间和核心体温（耳鼓膜温度）。*

**手术后/转出恢复室后：**

- 进入重症监护病房前的时间和核心体温（耳鼓膜温度），拔管时间，患者睁眼时间，PACU停留时间，是否保暖措施及持续时间，是否发生寒颤
- 术后的ICU住院时间和总住院时间
- 术后30天患者死亡率
- 术后30天手术切口感染率：术后30天内出现切口流脓或连续5天以上疼痛性红肿并使用抗生素治疗可判定为切口感染

# 研究统计学考虑及统计分析计划

## 样本量计算和抽样方法

### 样本量计算

根据文献报道13，术后进入ICU时低体温的发生率为45%，根据现场调查的样本量估计公式N=PQ/(d/t)^2^，P为低体温发生率，取值0.45，Q=1-P=0.55，d为可容许误差，一般取P值的10%左右，t为显著性检验的统计量，估计所需患者489例。考虑到是整群抽样及各个医院之间的均衡，在估计样本的基础上，再增加50%，拟入组800名患者。

### 抽样方法：采用多级抽样方法

把医院分按照级别（二级和三级）进行分层。采用整群抽样方法确定被调查医院，拟入选24家医院，详细计划如下：

| 分层 | 至少收集病例数 | 拟抽取的医院数 | 拟抽取的总病例数 |
| --- | --- | --- | --- |
| 二级医院 | 20例/医院 | 8家 | 160例 |
| 三级医院 | 40例/医院 | 16家 | 640例 |
| 总计 | ---- | 24家 | 800例 |

采用单纯随机抽样从每家医院抽取所需的例数。

具体实施方法：各研究中心在手术开始前1天（至少提前1天）将该中心全部择期全麻手术的排班表按照入排标准进行筛选,并将筛选后合格的排班表发送至CRC, CRC确认无误后将排班表编号后发送至统计师，由统计师按照统一标准进行随机抽样，生成随机抽样表，各中心按照由统计师发回的随机抽样结果进行受访者入组。

### 总体患者基线数据收集：

为了检验随机抽样人群的代表性，在研究过程将收集所有研究期间所有符合该研究入选排除标准的基线数据进行样本代表性的比较并了解该地区手术类型的分布情况。


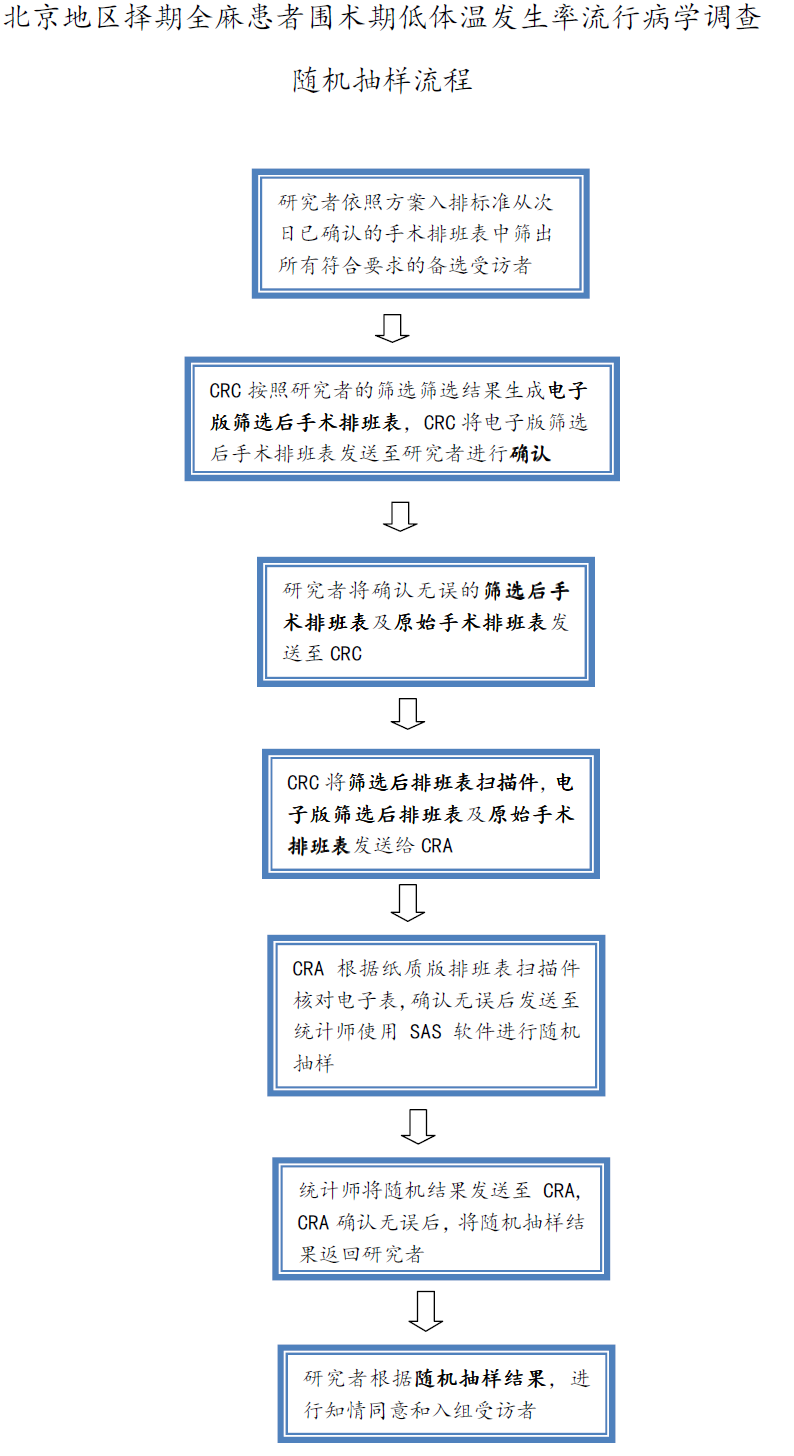


## 数据管理和统计分析

### 数据管理

1. 病例报告表（CRF）

研究者应根据受访者的原始观察记录，及时、完整、正确、清晰地填写CRF。

CRF不应涂改，如果确有错误需要修改，应当在修改处签名（见CRF填写说明）。

1. 建立数据库

数据管理员在进行数据录入前，要了解观察表格中各项目的内容及编码情况，将编码工作过程记录于编码本保存。数据库命名应规范、易读、易查找。并保证其正确、安全和保密。

1. 数据的录入与修改

采用EPIDATA3.1软件编制数据录入程序，进行数据录入与管理。为保证数据的准确性，应由两个数据管理员独立进行双份录入并校对。对病例报告表中存在的疑问，数据管理员将写入疑问解答表(DRQ)，并通过临床监查员向研究者发出询问，研究者应尽快解答并返回，数据管理员根据研究者的回答进行数据修改、确认与录入，必要时可以再次发出DRQ。DRQ应保存备查。

### 统计分析

1. 一般原则

统计软件：SAS 9.2

定量指标的描述将计算例数、均数、标准差、中位数、最小值、最大值。分类指标的描述用各类的例数及百分数。

所有统计分析将采用SAS 9.2统计分析软件编程计算。所有的统计学检验均采用双侧检验，P值< 0.05将被认为所检验的差别有统计意义（特别说明的除外），可信区间采用95%的可信度。

1. 统计分析

失访分析：主要采用描述性分析，计算失访人群占总预计样本量的百分比。

低体温发生率：发生低体温的患者数/被调查的总数×100%

高危因素分析：

单因素分析：对年龄（<14岁和>60岁）、ASA分级>II级、BMI、麻醉类型（合并硬膜外和全麻是高危因素）、手术分级、手术持续时间（2小时或小于2小时）、麻醉持续时间（2小时或小于2小时)、手术类型、手术室温度、术中输/血液量及是否加温后使用、术中冲洗液量及是否加温后使用、术中输血量及是否加温后使用、是否采取保温的措施、麻醉用药的种类、不同保暖措施等进行比较分析，确定高危因素。

多因素分析：采用Logistic多元回归分析，对单因素的高危因素进行多元回归分析，确定各高危因素的影响程度。

术后体温变化的曲线拟合：推求一个解析函数y=f(x)使其通过或近似通过有限序列的体温点(xi，yi)，通常用多项式函数通过最小二乘法求得此拟合函数。

# 受访者完成及退出研究

## 受访者完成研究

受访者按照研究要求完成计划安排的规定的所有访视内容，则视为完成研究。受访者在研究过程死亡视为完成研究。

## 受访者退出研究

任何进入研究的受访者(已签署知情同意书)由于任何原因未能按照上述定义的要求完成研究, 将被视为退出研究。

受访者可能由于以下原因在研究结束之前终止研究：

- 自愿退出；
- 失访；
- 违反研究方案；
- 研究牵头单位要求终止研究；
- 其他

应尽可能的随访退出研究的患者。对于在手术后退出的患者应尽量随访之术后30天以尽可能收集所关注的临床研究结局，成功入选后的所有受访者的病例报告表均要填写完整，并且在病例报告表的“研究结论页”上记录所有被随机但未完成研究的受访者退出原因。

## 筛选和基线评估失败

患者在随机抽样获得知情同意之前退出研究被视为筛选失败。退出研究的原因需记录在患者的筛选/入选表中。

# 质量控制与保证

## 方案偏离

研究者有责任确保临床研究的执行不违背方案，除非在必须偏离方案以保护患者的生命或身体健康的紧急医疗情况下。任何偏离方案的事件，都应填写方案偏离病例报告表。北京协和医院将监控方案偏离的发生情况,以评估研究者对方案的依从性。

方案偏离包括但不局限于以下情况：

- 未遵循知情同意过程
- 未遵循随机化抽样原则
- 不满足方案入排标准
- 未能在时间窗内进行方案要求的临床随访

发生任何方案偏离事件时，研究者应被告知该研究中心的非依从行为。如果必要应采取纠正措施。如果经过二次培训仍然发生方案偏离，可能导致该中心的研究终止。

## 培训

北京协和医院负责对研究者和适当的研究参与人员提供培训和指导。在患者入组前，需对所有的研究者进行方案和临床研究操作程序的培训。研究者负责确保其指定的研究人员已得到对方案和研究过程的全面培训。

北京协和医院的监查员或委派者也应该进行方案、病例报告表或其他相关的研究程序与过程的适当培训。

## 法规依从性

本研究将按照方案和所有适用的中国法规要求进行。

## 研究监查

### 研究中心的监查

将制定并执行一份专门的研究监查计划，以确保临床研究符合方案的依从性。

### 依从性评估

北京协和医院/委派者可定期在不同研究中心进行依从性评估。在依从性评估期间，北京协和医院/委派者可以请求查阅和复制所有研究记录包括源文件。研究者和研究协调者在依从性评估过程中必须可以在场回应所有合理的要求和疑问。

# 数据

临床研究期间，研究者将保存完整准确的文件，包括但不限于以下内容：医疗记录、研究过程记录、实验室报告、病例报告表、签署的知情同意书、与伦理委员会和研究监查员通信、有关患者退出或研究结束的信息。

## 源文件

患者记录中应包括以下材料：

- 患者入组前的病史/身体状况
- 入组该研究当天起的记录，包含日期和签名、方案相关数字、研究中心和患者的信息，以及签署的知情同意书
- 每位患者的访视记录，包含日期和签名（适用于特定程序和检查结果）
- 报告的心血管事件、死亡事件、结果及支持性文件

# 病例报告表的完成

每个研究中心接受了方案和病例报告表填写培训的研究协调员将准确收集原始数据。北京协和医院/委派者将提供临床监查和病例报告表的核查，包括对源文件的核对。操作者工作表和医疗机构的医疗记录应与病例报告表的文件一起保存，需要时，北京协和医院/委派者可以使用以上文件。

# 伦理学考虑

## 伦理委员会审查

研究开始前，研究者需获得伦理委员会（EC）对方案和知情同意书的批准。未经伦理委员会、北京协和医院和/或监管机构的相应批准，方案或知情同意书不得更改。如果需要更改，任何方案修正和/或相关知情同意书的更新在执行前都应提交给伦理委员会并获得其书面批准。

## 知情同意

经过方案培训的研究者或委派者负责解释研究的性质和范围，并回答患者的疑问。随机分组前，所有的患者或其法定授权代理人必须签署由伦理委员会批准的知情同意书并注明日期。在签字时，必须评估患者并确认患者未受到影响其思维的药物的影响。

未能获得知情同意书的患者将不能参与本研究。签署的知情同意书将保存在患者的医疗记录中并提供给患者或其法定授权代理人一份副本。知情同意书的语言应满足国家食品药品监督管理局的要求并符合任何适用的法规。

## 保密性

为确保遵守国家食品药品监督管理局和任何适用的私人信息保护法规，整个研究过程中，各方应维持受保护的医疗信息的保密性。所有的数据将拒绝未经授权的访问。送往北京协和医院的病例报告表和其他研究记录上,将对每位患者分配一个唯一的编码进行识别。

# 技术路线图

低体温发生率的计算及分析

随机抽样

建立数据库，资料整理分析

总结报告

确定干预研究现场

现场调查

# 计划进度

## 研究持续时间

预计6个月，2013年5月-2013年11月

## 研究进度

2013年5月：确定调查方案，编写/印刷调查表；编写调查手册，培训调查员。

2013年6-7月：选择和落实干预现场，建立核心领导小组、组织工作机构和网络。

2013年8月：开展问卷调查及过程控制。

2013年10月：回收所有数据，并对数据进行整理、录入分析和统计。

2013年11月：撰写调查报告。

# 附录A

**美国麻醉医师协会(ASA) “术前病情评估标准”**

- **ASA病情评估1级**-健康情况正常的患者
- **ASA病情评估2级**-有轻度系统性疾病的患者
- **ASA病情评估3级**-有严重系统性疾病的患者
- **ASA病情评估4级**-存在持续威胁生命的严重系统性疾病的患者
- **ASA病情评估5级**- 必须接受手术才能生存的患者
- **ASA病情评估6级**-已被宣布脑死亡的即将捐献器官的患者

Source: <http://www.asahq.org/clinical/physicalstatus.htm>

# 附录B

**麻醉后寒战（PAS）分级**

PAS (Postanesthetic shivering)是指麻醉后出现的寒战.

按照Crossley和Mahajan描述的标准可以对麻醉后寒战的强度进行分级：

- **0** = 无寒战；
- **1** = 除了立毛，外周血管收缩，或两者同时存在（已排除其它原因），未见明显肌肉活动；
- **2** = 肌肉活动仅局限于一组肌肉群；
- **3** = 超过一组肌肉群存在中度肌肉活动但无大面积抖动；
- **4** = 全身肌肉剧烈运动。

# 附录C

**手术分级标准**

An operation represents a physiological stress. The magnitude of the physiological stress increases with the ‘invasiveness’ of the procedure. We have not been able to identify any widely accepted and validated system for classifying the stressfulness of operative procedures. We have therefore adopted a simple graded scale, which we have illustrated with examples.

Grade 1 (minor) Excision of lesion of skin; drainage of breast abscess

Grade 2 (intermediate) Primary repair of inguinal hernia; excision of varicose vein(s)

of leg; tonsillectomy/adenotonsillectomy; knee arthroscopy

Grade 3 (major) Total abdominal hysterectomy; endoscopic resection of prostate;

lumbar discectomy; thyroidectomy

Grade 4 (major+) Total joint replacement; lung operations; colonic resection;

radical neck dissection; neurosurgery; cardiac surgery

# 附录D

**手术切口感染判定标准（摘自美国CDC手术切口感染指南1999年版）**


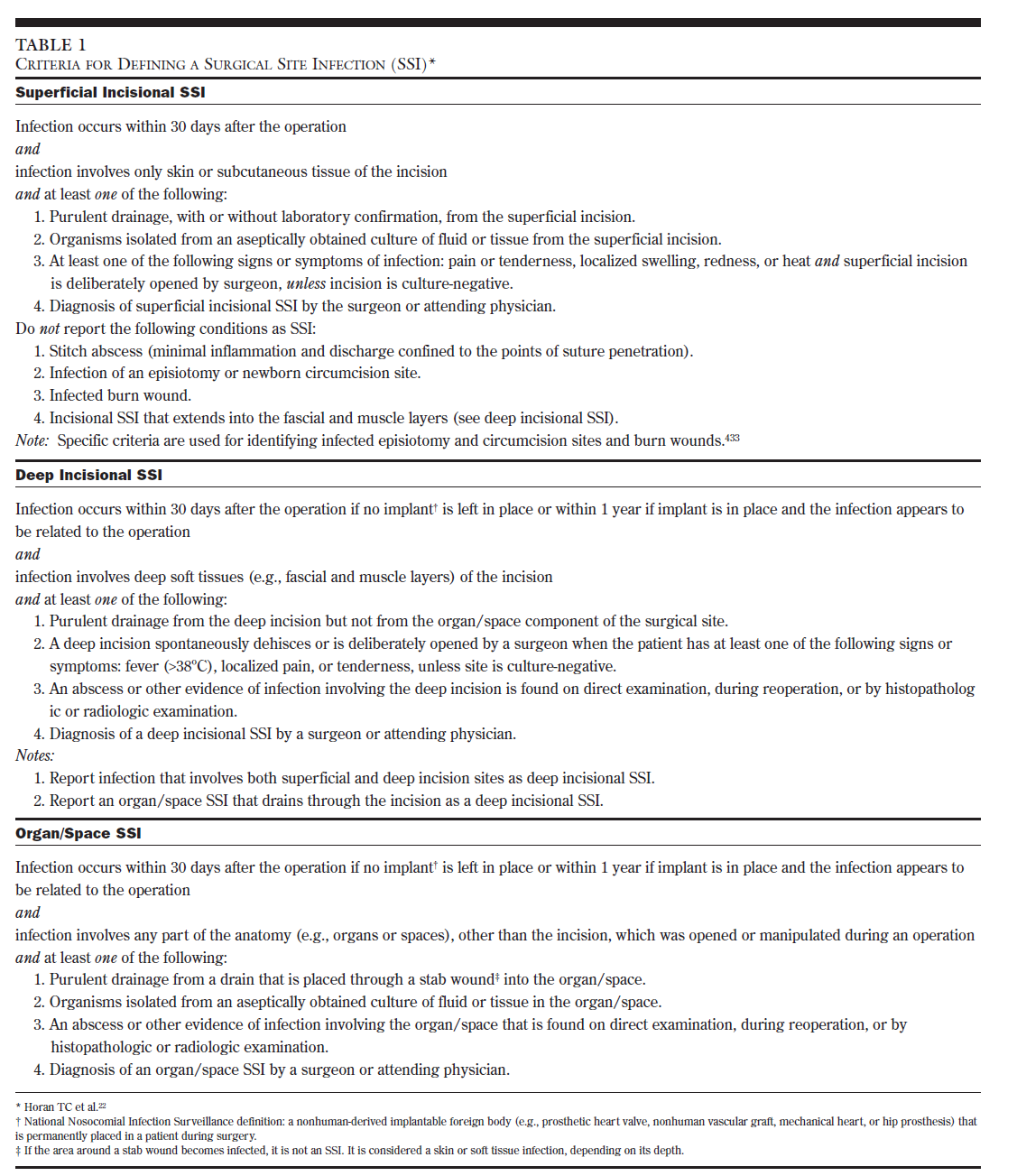


# 附录E

**参考文献**

1. Abelha FJ, Castro MA, Neves AM, Landeiro NM, Santos CC. Hypothermia in a surgical intensive care unit. BMC Anesthesiol 2005; 5: 7.

2. Frank SM, Beattie C, Christopherson R et al. Unintentional hypothermia is associated with postoperative myocardial ischemia. The Perioperative Ischemia Randomized Anesthesia Trial Study Group. Anesthesiology 1993; 78: 468–476.

3. Frank SM, Fleisher LA, Breslow MJ et al. Perioperative maintenance of normothermia reduces the incidence of morbid cardiac events. A randomized clinical trial. JAMA 1997; 277: 1127–1134.

4. Schmied H, Kurz A, Sessler DI, Kozek S, Reiter A. Mild hypothermia increases blood loss and transfusion requirements during total hip arthroplasty. Lancet 1996; 347: 289–292.

5. Kurz A, Sessler DI, Lenhardt R. Perioperative normothermia to reduce the incidence of surgical-wound infection and shorten hospitalization. Study of Wound Infection and Temperature Group. N Engl J Med 1996; 334:1209–1215.

6. Torossian A, Ruehlmann S, Middeke M et al. Mild preseptic hypothermia is detrimental in rats. Crit Care Med 2004; 32: 1899–1903.

7. Kongsayreepong S, Chaibundit C, Chadpaibool J et al. Predictor of core hypothermia and the surgical intensive care unit. Anesth Analg 2003; 96: 826–833.

8. Panagiotis K, Maria P, Argiri P, Panagiotis S. Is postanesthesia care unit length of stay increased in hypothermic patients? AORN J 2005; 81: 379–392.

9. Doufas AG. Consequences of inadvertent perioperative hypothermia. Best Pract Res Clin Anaesthesiol 2003; 17:535–549.

10. Mahoney CB, Odom J. Maintaining intraoperative normothermia: a meta-analysis of outcomes with costs. AANA J 1999; 67: 155–163.

11. Smith JJ, Bland SA, Mullett S. Temperature – the forgotten vital sign. Accid Emerg Nurs 2005; 13: 247–250.

12. Sessler DI. Complications and treatment of mild hypothermia. Anesthesiology 2001; 95: 531–543.

13. Young, V. Watson, M. Prevention of Perioperative Hypothermia in Plastic Surgery. Aesthetic Surgery Journal. 2006; 551-571.
