# Supplementary material for: Incidence of Inadvertent Intraoperative Hypothermia and Its Risk Factors in Patients Undergoing General Anesthesia in Beijing: A Prospective Regional Survey
Source: PLoS One. 2015 Sep 11;10(9):e0136136. doi: 10.1371/journal.pone.0136136 (PMC4567074; doi:10.1371/journal.pone.0136136)
Supplement: S2 Protocol — (DOCX) [file pone.0136136.s003.docx]

English Translation Version

Protocol Number: MDI0091

**An** **Epidemiology Survey on the Current Incidence Rate of Hypothermia during Perioperative Period of Elective Operations under General Anesthesia**

| **Principal Investigator:** | Huang, Yuguang |
| --- | --- |
| **Sponsor:** | Anesthesia Department, Peking Union Medical College Hospital, Chinese Academy of Medical SciencesBeijing Anesthesia Association, Chinese Medical Association |
| **Date of the Protocol Version** | April 15, 2013 |

**Study summary**

| Study Title: | An Epidemiology Survey on the Current Incidence Rate of Hypothermia during Perioperative Period of Elective Operations under General Anaesthesia |
| --- | --- |
| Objectives: | - To understand and grasp the incidence rate of hypothermia during perioperative period of elective operations under general anaesthesia in Beijing - To carry out a subgroup analysis on the survey data and explore the high-risk factors for the incidence of hypothermia |
| Study Sites: | Hospitals higher than grade two in Beijing (it is planned to randomly select 8 Level-2 hospitals and 16 Level-3 hospitals) |
| Target Population: | Candidates for an elective operation under general anesthesia |
| Inclusion Criteria | 1. Male or female, no limitation on age 2. Candidates for an elective operation under general anesthesia 3. The duration of operation is not expected over 30 minutes 4. The subject agreed to participate in the study and signed the informed consent |
| Exclusion Criteria | 1. Central high fever, including that induced by cerebrovascular disease, cerebral trauma, cerebral surgeries, epilepsy and acute hydrocephalus 2. Thermoregulation abnormalities including malignant hyperthermia (MHS) and neuroleptic malignant syndrome 3. Infectious fever 4. Patients confirmed diagnosed with hypothyroidism or hyperthyroidism 5. Patients whose preoperative core temperature was higher than 38.5 °C |
| Sample Size: | According to the previous reports in references, the incidence rate of hypothermia was about 45%, according to the formula for estimating the sample size of onsite survey N=PQ/(d/t)2, P was the incidence rate of hypothermia, which was set as 0.45; Q=1-P=0.55, d was the tolerance, which was normally about 10% of P value; t was the statistic for the significance test, for which an estimated number of 489 patients were required. Considering cluster sampling and the balance among different hospitals, another 50% was increased on the basis of the estimated sample, and thus 800 subjects were planned to be included. |
| Sampling Method: | Stratified cluster random sampling |
| Primary Indicator: | The incidence rate of hypothermia |
| Statistical Analyis Plan | 1. The incidence rate of hypothermia: the number of patients with hypothermia / the total number of patients for investigation × 100% 2. Multi-factor analysis: Logistic multiple regression analysis, the high risk factors for single factors were subjected to multiple regression analysis to determine the influence degree of the high risk factors. 3. Statistical modeling for curve fitting of postoperative body temperature changes. |

**1. Study background:**

Mild hypothermia during perioperative period is a very common, and the percentage accounts for about a half of all the patients for operations (1), which is directly related to the three major adverse events as below and significantly increases their risks: 1) cardiac complications; (2, 3), 2) hemorrhage (4), 3) infection complications (5, 6), particularly in patients with high risk factors (ASA grade III, IV) (7). Furthermore, the decrease in body temperature may also lead to the following situations: 1) prolongation in retention time in post-anesthetic ICU (PACU) (8), 2) decrease in the thermal comfort for patients (9), thus affecting the satisfaction degree of patients and increases the cost (10).

From the viewpoint of the safety of the patients, body surface temperature is only a physical sign (11). Therefore, in order to prevent hypothermia during perioperative period, core temperature monitoring must be carried out from the very beginning of the operation. Secondly, the heat loss during operations should be minimized; thirdly, initiative measurements for increasing temperature should be carried out for high risk patients during the entire procedures of operations (12).

Although the related positive effects for carrying out measurements to prevent hypothermia during perioperative period has been proved in most of the patients, no national practice guidance has been published until now. To obtain more information on body temperature monitoring and practices in keeping warm for patients in our country, we carried out the prospective survey on the samples from an anesthesia department in Beijing. By investigating the incidence rate of hypothermia during perioperative period in this area and exploring the reasons for hypothermia, the incidence rate of hypothermia during perioperative period was estimated on this basis, which provided evidence for carrying out measurements for keeping warm during operations.

**2. Objectives of the study**

2.1. To understand and grasp the current incidence rate of hypothermia during perioperative period in Beijing.

2.2. To carry out subgroup analysis on the survey data and explore the high risk factors for hypothermia.

2.3. To carry out curve fitting for the tendency of body temperature changes in the subjects after anesthesia.

**3. Study design and duration**

The present study was a cross-sectional study on the incidence rate of hypothermia during perioperative period of elective surgery under general anaesthesia in Beijing. The study observation stage was a previously selected week, and the cases for observations were randomly selected in the week and corresponding data were collected.

1. **Exposure of interest**

5.1 Risk factors for the patients:

Age (<14 and >60), ASA grading > grade II, BMI, diabetes, the preoperative basal core body temperature of the patient

5.2 Risk factors for anesthesia:

Type of anesthesia (complicated epidural anesthesia and non-complicated epidural anesthesia), category of anaesthetic, duration of anesthesia, height of vertebral column anesthesia

5.3 Operations and other risk factors

Operation grading, duration of operation (2 hours), operation type (endoscopic surgery and non-endoscopic surgery), temperature in the operation room

5.4 Other risk factors

Intraoperative infusion amount (>4000 ml) and whether it was used after heating, intraoperative rinsing solution amount and whether it was used after heating, intraoperative blood infusion amount and whether it was used after heating, whether measurements for keeping warm were taken and the type of the measurements for keeping warm.

1. **Major clinical outcome of the study (outcome of interest)**

2.1 All of the patients for investigation and the incidence rate of postoperative hypothermia in subgroups.

2.2 All of the patients for investigation and the incidence rate of preoperative, intraoperative (it was recorded once for every 15 minutes after the start of anesthesia), and postoperative hypothermia (before entering the ICU wards) in subgroups.

Hypothermia was defined as a core body temperature below 36 °C:

- - Mild: core temperature: 35-35.9°C
- Moderate: core temperature 34-34.9°C
- Serious: core temperature ≤ 33.9°C

The incidence rate of hypothermia: the number of patients with hypothermia / the total number of patients for investigation × 100%

3. The incidence rate of shiver in PACU after operations

4. The mortality within 12 hours after operations

1. **Study experimental methods and technical route**

7.1 To establish the organization for intervention and form the working network

On the basis of determining the study scene, the project group of Peking Union Medical College Hospital acted as the leader to establish the survey organization for intervention, including the responsible leaders and the directors of anesthesia department in different hospitals. Specific staffs were designated for each organization so as to form the working network.

7.2 To select the study scenes and subjects meeting the study requirements

7.2.1 Study scene: hospitals higher than grade two in Beijing were to be included in the present study.

7.2.2 Subjects: the elective operation patients in the form of general anaesthesia during the survey.

7.2.2.1 Inclusion criteria

7.2.2.1.1 Male or female, no limitation on age

7.2.2.1.2 Elective operation patients for general anaesthesia

7.2.2.1.3 It was anticipated that the duration of the operation was longer than 30 minutes

7.2.2.1.4 Acquisition of informed consent

7.2.2.2 Exclusion criteria

7.2.2.2.1 Central high fever, including central high fever induced by cerebrovascular disease, cerebral trauma, cerebral surgeries, epilepsy and acute hydrocephalus

7.2.2.2.2 Thermoregulation abnormalities including malignant hyperthermia (MHS) and neuroleptic malignant syndrome

7.2.2.2.3 Infectious fever

7.2.2.2.4 The core temperature of the patients one week before operation was higher than 38.5℃

7.2.2.2.5 Patients confirmed diagnosed with hypothyroidism or hyperthyroidism

7.3 Baseline

Before the patients participated in the present study, the procedure of informed consent should be carried out according to the specifications in GCP, and the informed consents from the patients should be obtained.

The data as below should be collected at baseline:

- Demographic data, including age, gender and race
- Medical history regressive analysis, physical examinations, including height and body weight
- According to the inclusion and exclusion criteria, evaluate whether the patients can be included in the present study.
- Core temperatures at operation waiting area and before anesthesia induction

7.4 Perioperative period and postoperative period

The patients should receive proper treatments according to the criteria in the practicing criteria for medical facilities in China.

The data as followed should be collected:

- Dosage of anesthesia, analgesia and conscious sedation
- Time for starting and terminating anesthesia
- Operation duration
- Core temperature was recorded every 15 minutes

Post-anesthctic ICU (PACU)

After the operations, the patients were transferred back to the recovery area, the investigators recorded the postanesthetic recovery time, shivering, nausea and vomiting of the patients during this period. The reference criteria for transferring the patents out of the recovery room were as follows:

1. The patients were conscious and reactive, and the symptoms of pain and nausea have been controlled;

2. The vital signs are stable (the systolic pressure was within 20% of the baseline level, and the heart rates ranged from 50 to 100 times /minute);

3. The hemoglobin concentration was stable when blood transfusion was not carried out;

4. The peripheral blood oxygen saturation was higher than 95% at spontaneously breathing;

5. Core temperature ≥36 ℃;


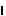


The time for meeting with the criteria for transferring out of the recovery rooms as above was recorded.

Operations / after transferring out of the recovery rooms:

- The duration in ICU
- The mortality of the patients within 12 hours after operation

1. **Statistical consideration and statistical analysis plan for the study**

8.1 Sample size calculation and sampling method

According to the report in reference 13, the incidence rate of hypothermia when the patients were transferred into ICU after operations was 45%, according to the formula for estimating the sample size of on-spot survey N=PQ/(d/t)2, P was the incidence rate of hypothermia, which was set as 0.45, Q=1-P=0.55, d was the tolerated error and it was normally about 10% of P value, t was the statistic for the significance test, for which an estimated number of 489 patients were required. Considering cluster sampling and the balance among different hospitals, another 50% was increased on the basis of the estimated sample, and thus 800 subjects were planned to be included.

The sampling method: the multi-grade sampling method was used

The hospitals were stratified according to their grades (grade two and grade three). The cluster sampling method was used to determine the hospitals for investigations, 24 hospitals were planned to be included, and the detailed protocol was shown as below:

| Stratification | Minimum collected cases required | Number of hospitals planned to be included | Total cases planned to be included |
| --- | --- | --- | --- |
| Grade two hospitals | 20 cases/ hospitals | 8 hospitals | 160 cases |
| Grade three hospitals | 40 cases/ hospitals | 16 hospitals | 640 cases |
| Total | --- | 24 hospitals | 800 cases |

The simple random sampling method was used, and the required case number was included in each hospital.

Specific implemented method: all the elective operations within a certain week were randomly selected.

8.2 Data management and statistical analysis

8.2.1 Data management

8.2.1.1 Case report form (CRF)

The investigators fill in the CRF timely, completely, correctly and clearly according to the original observation records.

CRF should not be smeared or modified, if modification is required due to mistakes, signature should be provided at modification (please refer to the descriptions for filling the CRF).

8.2.1.2 Database establishment

The data managing staffs should understand the contents and the coding of the items in the forms before data inputting, and the coding process was recorded in the coding book for preservation. The naming of the database should be standard, and it should be easy for reading and searching. The correctness, safety and confidentiality should be guaranteed.

8.2.1.3 Data inputting and modification

EPIDATA3.1 software was used for compile the inputting procedure for data inputting and management. In order to guarantee the correctness of the data, two data managing staffs should input and verify the data independently. Should they hold any question in the case report, the data managing staffs can fill them in the Data ReQuest (DRQ), and inquire the investigators via the clinical supervisors, the investigators should answer the questions and return the answers as soon as possible, afterwards the data managing staffs modify, confirm and input the data according to the answer of the investigators, and DRQ can be sent again if necessary. DRQ should be preserved for future reference.

8.2.2 Statistical analysis

8.2.2.1 General principles

Statistical software: SAS9.2

The number of cases, mean, standard deviation, median value, maximal value and minimal value should be calculated for the descriptions of the qualitative parameters. Number of cases and percentages are used for the descriptions on the classification parameters.

SAS9.2 statistical analysis software was used for the calculation of all the statistical analyses. Two side test is used for all the statistical tests, P<0.05 indicates that the difference is statistically significant (unless indicated otherwise), and 95% confidence is used as the confidence interval.

8.2.2.2 Statistical analysis

Analysis on loss of follow-ups: descriptive analysis is mainly used to calculate the percentage of lost cases in the totally anticipated sample size.

The incidence rate of hypothermia: the patients with hypothermia / the total number for survey ×100%

High risk factor analysis:

Single factor analysis: ages (<14 and >60), ASA grading > grade II, BMI, Type of anesthesia (complicated epidural anesthesia and non-complicated epidural anesthesia), grading of operations, duration of operations (2 hours or less than 2 hours), type of operations, temperature in the operation room, intraoperative infusion amount (>4000 ml) and whether it is used after heating, intraoperative rinsing solution amount and whether it is used after heating, intraoperative blood infusion amount and whether it is used after heating, measurements for keeping warm, category of anesthetic are compared.

Multiple factor analysis: Logistic multiple regression analysis is performed, and multiple regression analysis is carried out for the high risk factors for single factors to determine the influence degrees of the high risk factors.

Curve fitting for postoperative body temperature changes: an analytic function is deduced, y=f(x), to keep it passed or almost passed the body temperature point of finite sequence (xi, yi), and this fitting function is normally obtained by polynomial function using the least square method.

1. **Technical route**

Calculation and analysis of the incidence rate of hypothermia

Random sampling

Database establishment, data sorting and analysis

Summary report

Determination of the scene for intervention study

Onsite investigation

1. **Protocol schedule:**

It was estimated for six months, from March 2013 to July 2013

Study progress:

March 2013: to determine the survey protocol, compile / print questionnaire; train surveyors.

May 2013: to select and implement the scene for intervention, establish leading group, organize working mechanism and network.

June 2013: to carry out questionnaire survey and process control.

July 2013: to recover all of the data, sort, input, analyze and carry out statistics for the data.

August 2013: to draft the survey report.

**Appendix A: American Society of Anesthesiologists (ASA) “evaluation criteria for preoperative pathogenetic conditions”**

Grade 1 of ASA pathogenetic condition evaluation – patients with normal health conditions

Grade 2 of ASA pathogenetic condition evaluation – patients with mild systemic diseases

Grade 3 of ASA pathogenetic condition evaluation – patients with severe systemic diseases

Grade 4 of ASA pathogenetic condition evaluation – patients with persistent severe life-threatening systemic diseases

Grade 5of ASA pathogenetic condition evaluation – patients can only survive after operations

Grade 6 of ASA pathogenetic condition evaluation – patients had been announced as brain death and would donate their organs

Source: <http://www.asahq.org/clinical/physicalstatus.htm>

**Appendix B:** postanesthetic shivering (PAS) grading

PAS (Postanesthetic shivering) refers to the shivering after anesthesia.

The intensity of postanesthetic shivering can be graded according to the criteria described by Crossley and Mahajan:

0 = no shivering;

1 = besides piloerection, peripheral vaso-constriction is detected, or they are simultaneously detected (other reasons had been ruled out), but no obvious musculation is detected;

2 = musculation is confined to a group of muscle group;

3 = moderate musculation in more than one muscle group but trembling in large areas is not detected;

4 = heavy exercise of muscles all over the body.

Appendix C: operation grading criteria:

An operation represents a physiological stress. The magnitude of the physiological stress

increases with the ‘invasiveness’ of the procedure. We have not been able to identify

any widely accepted and validated system for classifying the stressfulness of operative

procedures. We have therefore adopted a simple graded scale, which we have illustrated

with examples.

Grade 1 (minor) Excision of lesion of skin; drainage of breast abscess

Grade 2 (intermediate) Primary repair of inguinal hernia; excision of varicose vein(s)

of leg; tonsillectomy/adenotonsillectomy; knee arthroscopy

Grade 3 (major) Total abdominal hysterectomy; endoscopic resection of prostate;

lumbar discectomy; thyroidectomy

Grade 4 (major+) Total joint replacement; lung operations; colonic resection;

radical neck dissection; neurosurgery; cardiac surgery

**APPENDIX D: Reference**

1. Abelha FJ, Castro MA, Neves AM, Landeiro NM, Santos CC. Hypothermia in a surgical intensive care unit. BMC Anesthesiol 2005; 5: 7.

2. Frank SM, Beattie C, Christopherson R et al. Unintentional hypothermia is associated with postoperative myocardial ischemia. The Perioperative Ischemia Randomized Anesthesia Trial Study Group. Anesthesiology 1993; 78: 468–476.

3. Frank SM, Fleisher LA, Breslow MJ et al. Perioperative maintenance of normothermia reduces the incidence of morbid cardiac events. A randomized clinical trial. JAMA 1997; 277: 1127–1134.

4. Schmied H, Kurz A, Sessler DI, Kozek S, Reiter A. Mild hypothermia increases blood loss and transfusion requirements during total hip arthroplasty. Lancet 1996; 347: 289–292.

5. Kurz A, Sessler DI, Lenhardt R. Perioperative normothermia to reduce the incidence of surgical-wound infection and shorten hospitalization. Study of Wound Infection and Temperature Group. N Engl J Med 1996; 334:1209–1215.

6. Torossian A, Ruehlmann S, Middeke M et al. Mild preseptic hypothermia is detrimental in rats. Crit Care Med 2004; 32: 1899–1903.

7. Kongsayreepong S, Chaibundit C, Chadpaibool J et al. Predictor of core hypothermia and the surgical intensive care unit. Anesth Analg 2003; 96: 826–833.

8. Panagiotis K, Maria P, Argiri P, Panagiotis S. Is postanesthesia care unit length of stay increased in hypothermic patients? AORN J 2005; 81: 379–392.

9. Doufas AG. Consequences of inadvertent perioperative hypothermia. Best Pract Res Clin Anaesthesiol 2003; 17:535–549.

10. Mahoney CB, Odom J. Maintaining intraoperative normothermia: a meta-analysis of outcomes with costs. AANA J 1999; 67: 155–163.

11. Smith JJ, Bland SA, Mullett S. Temperature – the forgotten vital sign. Accid Emerg Nurs 2005; 13: 247–250.

12. Sessler DI. Complications and treatment of mild hypothermia. Anesthesiology 2001; 95: 531–543.

13. Young, V. Watson, M. Prevention of Perioperative Hypothermia in Plastic Surgery. Aesthetic Surgery Journal. 2006; 551-571.
